# Amount, Source and Pattern of Dietary Protein Intake Across the Adult Lifespan: A Cross-Sectional Study

**DOI:** 10.3389/fnut.2020.00025

**Published:** 2020-03-16

**Authors:** Benoit Smeuninx, Carolyn A. Greig, Leigh Breen

**Affiliations:** ^1^School of Sport, Exercise and Rehabilitation Sciences, University of Birmingham, Birmingham, United Kingdom; ^2^MRC-Arthritis Research UK Centre for Musculoskeletal Ageing, Birmingham, United Kingdom; ^3^NIHR Birmingham Biomedical Research Centre, Birmingham, United Kingdom

**Keywords:** sarcopenia, nutrition, aging, skeletal muscle, protein

## Abstract

**Objectives:** Sub-optimal dietary protein consumption may partially underlie the age-related loss of muscle mass and function (sarcopenia). Specifically, dose, timing, source and distribution of dietary protein across the day might influence muscle anabolism in individuals from across the lifespan.

**Design:** The present study aimed to assess daily and meal-specific protein intake, protein source and protein intake pattern in 40 young (23.8 ± 4.3 years), 40 middle-aged (51.6 ± 4.1 years), and 40 old (77.4 ± 7.4 years) individuals using 3-day weighed food diaries.

**Results:** Old individuals consumed on average 83.4 ± 24.6 g of daily protein, which was significantly lower compared with young but not middle-aged individuals who consumed, respectively, 105.1 ± 43.0 g and 97.0 ± 31.1 g of daily protein (*P* = 0.013). No significant difference in daily protein intake was found with middle-aged individuals. Dietary protein intake pattern was uneven across meals for all groups (*P* < 0.001 for all). Sources of protein consumption were similar between groups except at lunch where old individuals ingested lower quality proteins compared with middle aged and young individuals.

**Conclusion:** Although total daily protein intake was sufficient in the majority of participants, per-meal protein intake and protein distribution contend the current knowledge regarding optimal protein intakes. Increasing protein intake, especially at breakfast and lunch, could mitigate age-related muscle loss.

## Introduction

The progressive decline in skeletal muscle mass and function observed with advancing age, termed sarcopenia, can lead to an increased risk of falls, frailty and mortality (1). Skeletal muscle maintenance is therefore a cornerstone for healthy aging. Changes in muscle mass are ultimately the product of the complex interplay between muscle protein synthesis (MPS) and muscle protein breakdown (MPB) (2). Dietary protein provision robustly stimulates MPS and, to a lesser extent, decreases MPB, resulting in net muscle protein accretion (3). However, an impaired muscle anabolic response to the ingestion of lower protein doses in old individuals (termed “anabolic resistance”), is thought to be a pivotal factor in sarcopenia (4, 5). Less well documented is the role of dietary protein on muscle mass regulation during middle-age, a potential transition period between normal and impaired muscle anabolic sensitivity. As muscle mass begins to noticeably decline from ~45 years of age (6), it is therefore important to understand dietary protein intakes/requirements at this stage of life, in order to identify an appropriate time at which to introduce dietary strategies to delay the consequences of sarcopenia.

The Recommended Dietary Allowance (RDA) for protein of 0.8 g·kg^−1^·day^−1^ is deemed adequate and meets the metabolic demands of this nutrient, irrespective of age and gender. However, the dietary protein RDA is thought to be insufficient for repeated, robust stimulation of MPS and, hence, maintenance of muscle mass in old adults. Indeed, higher protein intakes of 1.0–1.5 g·kg^−1^·day^−1^ are associated with increased muscle mass and strength in old individuals (7–9). The proposal that dietary protein requirements are higher in older age are reinforced by recent data demonstrating that older individuals require considerably more protein on a per-meal-basis to maximally stimulate MPS compared with their younger counterparts (0.4 vs. 0.24 g·kg^−1^) (10). This is problematic for many older individuals, who typically consume dietary protein unevenly across meals, with the majority of protein intake being consumed during one meal (11). This uneven pattern of daily protein intake likely results in a failure to meet the threshold for maximal MPS stimulation during most meals. Developing dietary protein strategies that enable maximal MPS stimulation with every meal may be essential for attenuating the progression of sarcopenia.

The source of dietary protein is an important determinant of postprandial MPS stimulation. Specifically, the AA profile and absorption/digestive properties of ingested protein may determine the extent of MPS stimulation. Proteins that elicit a rapid increase in blood aminoacidemia/leucinemia, generally stimulate MPS to a greater extent than proteins with slower digestive properties or an inferior AA/leucine profile (12). In a typical Western diet, protein consumption primarily originates from meat and dairy products. The majority of animal-derived proteins have an AA profile that closely matches the bodily requirements and, as such, are able to evoke greater MPS stimulation compared with plant-derived proteins (13). However, reductions in appetite brought on by physiological (impaired sensory perception, poor chewing capability) and psychosocial factors (loneliness, cost), make it challenging for many older individuals to consume sufficient protein with each meal (14, 15), particularly from animal sources (16). Therefore, more pragmatic approaches are required in order to develop feasible protein intake guidelines for older individuals, formulated on a meal-to-meal basis.

The dearth of evidence on dietary protein habits across the adult lifespan hampers the development of tailored dietary interventions to support skeletal muscle health in older age. Therefore, the purpose of the present study was to assess the habitual dietary intakes of healthy young, middle-aged and community-dwelling old individuals living in the UK, with a focus on the amount, source and pattern of intake.

## Methods

### Participants

Young (*n* = 40; 23 ± 4.3 years), middle-aged (*n* = 40; 51.6 ± 4.1 years), and old (*n* = 40; 77.4 ± 7.4 years) adults were recruited from the Birmingham area (West Midlands, UK). Participants were eligible if deemed healthy based on a general health questionnaire and ambulatory. Institutionalized or dependent living individuals were excluded from study participation. Group characteristics are presented in Table 1. All participants were informed of the study procedures and provided written consent to participate. Ethical approval was obtained through the University of Birmingham Research Ethics Committee (#13-1475A). The study conformed to the latest guidelines set by the Declaration of Helsinki (7th edition).

**Table 1:** Participant characteristics.

	**Young**	**Middle**	**Old**
Sample size (N)	40	40	40
Male/Female (N/N)	26/14	12/28	21/19
Age (y)	23.8 ± 4.3	51.6 ± 4.1^*^	77.4 ± 7.4^*^^†^
Weight (kg)	70.9 ± 11.9	74.1 ± 15.1	72.0 ± 12.7
Height (cm)	173.6 ± 8.3	168.2 ± 13.4	170.8 ± 8.6
BMI (kg·m^−1^)	23.5 ± 2.6	26.6 ± 6.2^*^	24.5 ± 3.0

* indicates a significant difference from young (P < 0.05),

† *indicates a significant difference from middle (P < 0.05). Values are presented as means ± SD*.

### Dietary Intake Recording

Participants' height and body mass were assessed in light clothing to the nearest 0.1 cm and 0.1 kg, respectively, using a stadiometer and digital scales. Participants were given a 3-day weighed food diary to be completed over 2 week-days and 1 weekend-day. The food diary required participants to provide the time, preparation method, brand and weight of all food ingredients and drinks consumed. Participants were given written and verbal instructions on how to accurately complete the food diary. Kitchen scales (Wuwangni, WeiHeng, Hong Kong) were provided to accurately determine the weight of foods and drinks ingested.

### Nutritional Data Analyses

Weighed food diaries were analyzed using Dietplan 7 software (Forestfield Ltd, West Sussex, UK, V7.00.46). Daily total energy intake (TEI), macronutrient and micronutrient composition data were generated. Dietary macronutrient values were calculated relative to participants' body mass (g·kg^−1^), and as a relative percentage of total daily energy intake. Relative protein intakes were compared with the current RDA for protein consumption (0.8 g·kg^−1^·day^−1^), and with an alternative recommendation for higher protein in older individuals (1.0 g·kg^−1^·day^−1^) (8). Daily dietary intake was divided into 4 time points; T1, T2, T3, and T4, respectively corresponding to breakfast (6.00–10.00 h), lunch (11.30–14.30 h), dinner (17.30–22.00 h), and snacks (remainder of the day outside T1, T2, and T3). Relative protein intake at each meal was compared against the 0.24 and 0.40 g·kg^−1^ thresholds for maximal MPS stimulation for young and old individuals, respectively (10), and used to assess the proportion of meals that reached these respective thresholds. Furthermore, participants' daily protein intake pattern for T1, T2, and T3 was determined. The meal with the highest relative protein content was given a score of 3 and used as a reference value. Meal protein intake differed from the reference meal if a 10–20% (score 2) or >20% (score 1) decrease was observed in relative protein intake. This resulted in 18 distinct protein intake patterns. Finally, the protein source contributing the highest absolute amount of protein at each meal time-point was determined for all participants, and presented as a percentage of individuals consuming the respective protein source. Most commonly identified protein sources included: bread, red meat, poultry/eggs, milk, cheese, yogurt, protein supplements, fish, nuts, soya, vegetarian meat substitutes and oats/muesli.

### Statistical Analysis

Sample size was determined using point estimate calculations for protein intake, with a population mean based on a study by Cardon-Thomas et al. (11), to allow a margin for random error of 0.05 g·kg^−1^·day^−1^. Data were analyzed using Graphpad Prism V7.0 (Graphpad Software, CA, USA). Between- group differences for total nutrient intakes and participant characteristics were assessed using an ordinary 1-way ANOVA. Within and between-group differences for meal-specific protein intakes were identified using an ordinary 2-way ANOVA. Tukey *post-hoc* analyses were used. *F*-values represent the ratio of systematic to unsystematic variation, with a value greater than one indicating an effect beyond extraneous factors. Spearman's correlations were used to determine all associations between protein intake (absolute and relative) and daily total energy intake (TEI), with the exception of the correlation between absolute protein intake and TEI in the old group where a Pearson correlation was used. A binomial test was used to determine whether differences within groups for protein intake pattern (even or uneven intake) existed. A Fisher's exact test was used to determine whether protein intake pattern (even or uneven) differed between groups. Significance was set at *P* < 0.05. All values are expressed as mean ± SD unless stated otherwise.

## Results

### Dietary Energy and Macronutrient Intake

**Figure 1:**
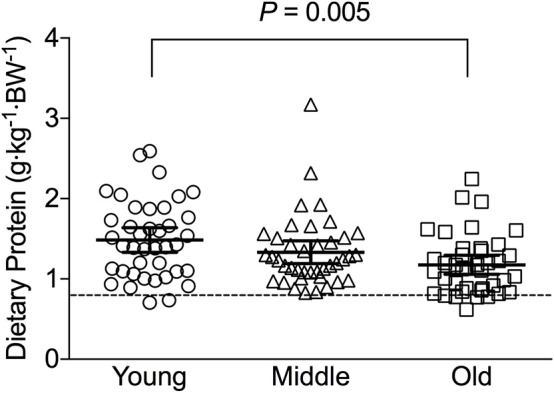
Average relative daily protein intake in young, middle-aged and older individuals. Scatter plots display mean with 95% confidence intervals. Dashed line indicates current recommended daily allowance for dietary protein allowance of 0.8 g·kg^−1^·day^−1^.

Average daily energy and macronutrient intakes are presented in Table 2. No differences between groups were found for TEI, or absolute and relative CHO, fat and alcohol consumption. Absolute (*P* = 0.013) and relative (*P* = 0.005) daily protein intakes were 26% higher in young compared with old individuals. Expressed as a percentage of TEI, old individuals consumed less protein and alcohol compared with young (*P* = 0.01 for both) and middle-aged individuals (*P* = 0.034 and *P* < 0.001, respectively), but more CHO compared with middle-aged individuals (*P* = 0.001). Absolute and relative daily protein intake in young and old individuals was positively associated with TEI, whereas only absolute protein intake showed a positive correlation with TEI in middle-aged individuals (Table 3). Over the entire 3-day measurement period, 95, 100, and 98% of young, middle-aged and old individuals, respectively, met the current RDA for protein intake of 0.8 g·kg^−1^·day^−1^ (Figure 1). However, only 70, 62, and 65% of, respectively, young, middle-aged and old individuals met this threshold on all 3 individual measurement days. When compared to the alternative protein recommendation of 1.0 g·kg^−1^·day^−1^, a significantly greater proportion of young individuals (60%) reached this protein intake on all 3 individual measurement days as opposed to middle-aged and old individuals (both 35%) (*P* = 0.034).

**Table 2:** Average daily energy and macronutrient intakes in young, middle and old.

	**Young**	**Middle**	**Old**
Total energy intake (kcal)	2,257 ± 576.5	2,181 ± 606.9	2,169 ± 496.6
Total protein intake (g)	105.1 ± 43.0	97.0 ± 31.1	83.4 ± 24.6^*^
Protein intake (g·kg^−1^)	1.5 ± 0.5	1.3 ± 0.4	1.2 ± 0.4^*^
Protein (% TEI)	18.6 ± 6.4	18.1 ± 4.4	15.4 ± 3.0^*^^†^
Total CHO intake (g)	236.4 ± 73.6	211.1 ± 64.2	231.5 ± 64.6
CHO intake (g·kg^−1^)	3.4 ± 1.1	2.9 ± 0.9	3.3 ± 1.1
CHO (% TEI)	43.8 ± 6.4	41.4 ± 8.4	47.3 ± 6.3^†^
Total fat intake (g)	85.7 ± 23.4	86.7 ± 34.6	88.8 ± 25.0
Fat intake (g·kg^−1^)	1.2 ± 0.3	1.2 ± 0.5	1.3 ± 0.4
Fat (% TEI)	34.2 ± 5.8	35.1 ± 6.6	36.9 ± 6.4
Total alcohol intake (g)	11.6 ± 17.6	17.3 ± 21.7	9.3 ± 8.9
Alcohol (g·kg^−1^)	0.2 ± 0.2	0.2 ± 0.3	0.1 ± 0.1
Alcohol (% TEI)	3.5 ± 5.0	5.4 ± 6.4	0.4 ± 0.4^*^^†^

* indicates a significant difference from young (P < 0.05),

† *indicates a significant difference from middle (P < 0.05). Values are presented as means ± SD*.

**Table 3:** Correlations between protein intake (absolute and relative) and total energy intake (TEI), and between protein intake (absolute and relative) and age.

	***r***	***r*^**2**^**	***P***
Young TEI – Protein Intake (g·kg^−1^)	0.636	0.404	<0.001
Young TEI – Protein Intake (g)	0.505	0.255	<0.001
Middle TEI – Protein Intake (g·kg^−1^)	0.515	0.265	0.001
Middle TEI – Protein Intake (g)	0.290	0.084	0.069
Old TEI – Protein Intake (g·kg^−1^)	0.765	0.585	<0.001
Old TEI – Protein Intake (g)	0.643	0.413	<0.001
Age – Protein Intake (g·kg^−1^)	−0.288	0.082	0.001
Age – Protein Intake (g)	−0.349	0.122	<0.001

### Dietary Protein Distribution

**Figure 2:**
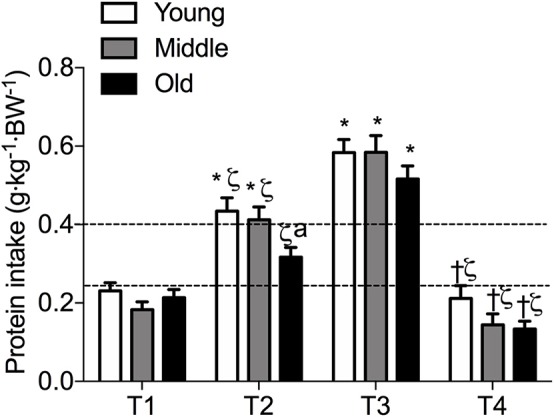
Meal-specific average protein intakes in young, middle-aged and older individuals. Dashed lines represent threshold protein intakes of 0.24 and 0.4 g·kg^−1^, suggested for maximal stimulation of MPS in young and older individuals, respectively. *indicates significantly different from T1, ζ indicates significantly different from T3, ^†^indicates significantly different from T2, ^a^indicates significantly different from young at same time-point. Significance was set at *P* < 0.05.

**Figure 3:**
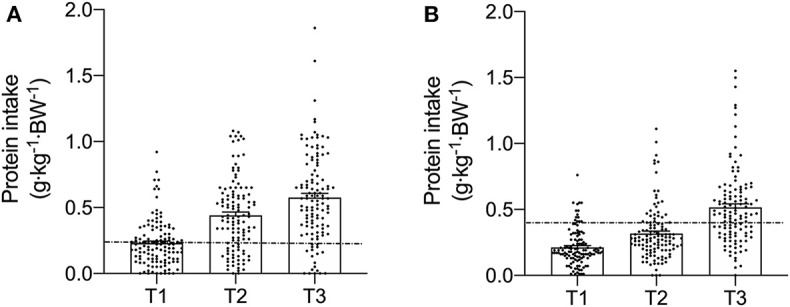
Meal-specific relative protein intakes for each meal consumed at breakfast (T1), lunch (T2), and dinner (T3) in young **(A)** and old **(B)**. Dashed lines represent threshold protein intakes of 0.24 and 0.4 g·kg^−1^, suggested for maximal stimulation of MPS in young and older individuals, respectively.

Meal-specific relative protein intakes are presented in Figure 2. Daily dietary protein intake was distributed unevenly across meals with ~16, 30, 39, and 15% of protein in young, ~14, 31, 44, and 11% of protein in middle-aged and ~8, 12, 75, and 5% of protein in old individuals being consumed at T1, T2, T3, and T4, respectively. A significant main effect for time and group was found for relative [*F*_(3, 468)_ = 115.53 and *F*_(2, 468)_ = 5.72, respectively] and absolute [*F*_(3, 468)_ = 107.31 and *F*_(2, 468)_ = 5.91, respectively] protein intake. No significant interaction effects were found for both relative [*F*_(6, 468)_ = 1.13] and absolute protein intake [*F*_(6, 468)_ = 1.31]. Relative and absolute protein intake was higher at T3 (dinner) compared with all other time-points for all groups (*P* < 0.01 for all). Greater relative and absolute protein intakes were observed at T2 compared with T1 in young and middle-aged individuals (*P* < 0.001 for both). Absolute and relative protein intakes were similar at T1 and T4 for all groups. Between-group differences in relative and absolute protein intake were found at T2 (lunch) only, where absolute protein intake was lower in old compared with young (*P* = 0.01) and middle-aged individuals (*P* = 0.03), and relative protein intake was lower in old compared with young (*P* = 0.01). On a meal-to-meal basis, the proposed dietary protein threshold for maximal MPS (0.24 g·kg^−1^ in young and 0.40 g·kg^−1^ in old) was met on all 3 measurement occasions by 28, 50, and 75% of young and 7.5, 7.5, and 30% of old individuals at T1, T2, and T3, respectively (Figures 3A,B). Snacks were often not consumed as a single-meal; therefore, it was not possible/appropriate to determine whether dietary protein MPS thresholds were met at T4 for young, middle-aged and old.

### Dietary Intake Protein Patterns

**Figure 4:**
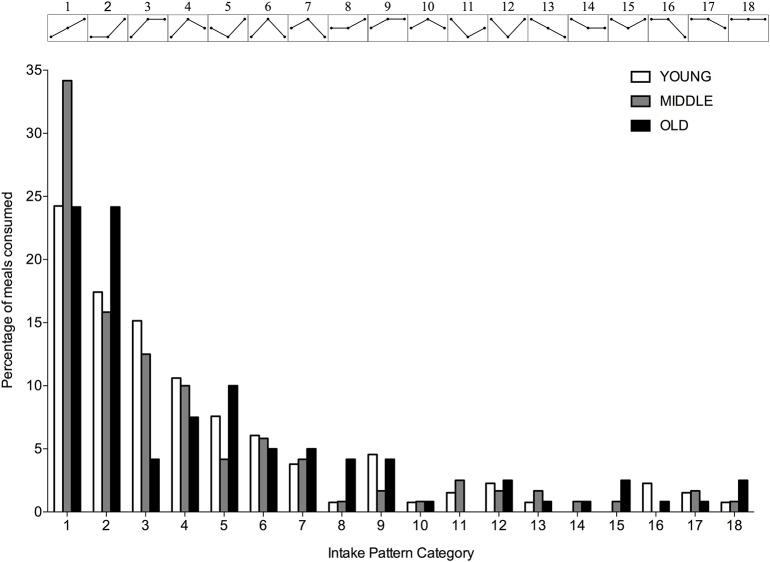
Percentage of young, middle-aged and older individuals for each observed protein intake pattern. Intake patterns are depicted above each bar and represent the relationship between protein intake at T1, T2, and T3, respectively.

A significantly greater proportion of young, middle-aged and old individuals displayed an uneven protein intake pattern (*P* < 0.001 for all) with no differences between groups (*P* = 0.617). The uneven protein distribution across the day, resulted in 18 observed protein intake patterns (Figure 4). Approximately 67, 63, and 53% of, respectively, young, middle-aged and old individuals distributed their daily protein intake according to one of three most frequently observed intake patterns. In young and middle-aged individuals, 72% of the meals highest in protein were consumed at T3, whereas 76% of the meals highest in protein were consumed at T3 in old individuals.

### Sources of Dietary Protein Intake

At T1 and T4, milk was the most common source of protein intake for all three groups. At T2, young and middle-aged individuals primarily consumed animal-derived proteins in the form of poultry, fish and red meat, whereas plant-based proteins (bread) were the main source of protein in old individuals. At T3, animal-based proteins were most commonly consumed by all three groups. Table 4 represents the 5 most commonly consumed protein sources for each meal in young, middle-aged and old individuals.

**Table 4:** Top 5 most commonly consumed protein sources during breakfast (T1), lunch (T2), dinner (T3), and snacks (T4) in young, middle-aged and old.

**Young**				**Middle**				**Old**			
**T1**	**T2**	**T3**	**T4**	**T1**	**T2**	**T3**	**T4**	**T1**	**T2**	**T3**	**T4**
Milk (25%)	Poultry (35%)	Meat (red) (37.5%)	Milk (32.5%)	Milk (47.5%)	Poultry (30%)	Poultry (42.5%)	Milk (50%)	Milk (67.5%)	Bread (32.5%)	Meat (red) (32.5%)	Milk (65%)
Bread (17.5%)	Fish (22.5%)	Poultry (37.5%)	Protein supplement (17.5%)	Poultry (25%)	Fish (27.5%)	Meat (red) (32.5%)	Yogurt (10%)	Bread (12%)	Fish (22.5%)	Poultry (30%)	Bread (5%)
Poultry (15%)	Meat (red) (20%)	Fish (7.5%)	Cake (12.5%)	Yogurt (10%)	Meat (red) (17.5%)	Fish (17.5%)	Bread (7.5%)	Yogurt (7.5%)	Poultry 15%)	Fish (27.5%)	Cake (5%)
Oats (12.5%)	Bread (15%)	Pizza (7.5%)	Poultry (7.5%)	Bread (5%)	Bread (10%)	Yogurt (2.5%)	Cake (7.5%)	Oats (5%)	Meat (red) 10%	Vegetarian substitute (2.5%)	Chocolate (5%)
Yogurt (12.5%)	Milk (2.5%)	Vegetarian substitute (5%)	Meat (red) (5%)	Oats (5%)	Cheese (7.5%)	Vegetarian substitute (2.5%)	Nuts (7.5%)	Poultry (5%)	Milk (7.5%)	Bread (2.5%)	Yogurt (2.5%)

*Percentages represent the fraction of individuals consuming the respective protein source*.

## Discussion

The present study is, to our knowledge, the first to directly compare dietary habits in young, middle-aged and old individuals with a focus on the amount, pattern and source of dietary protein intake. Absolute and relative protein intake was lower in old compared with young individuals. The RDA for protein (0.8 g·kg^−1^·day^−1^) was met by a majority of young, middle-aged and old individuals, whereas the number of old and middle-aged individuals (35%) meeting the proposed alternative higher protein RDA (1.0 g·kg^−1^·day^−1^) on all 3 measurement days was lower than young individuals (60%). Further to this, an uneven pattern of dietary protein intake was observed across meals for all groups, which was likely insufficient to reach the proposed threshold for maximal MPS stimulation at each meal in old, and potentially middle-aged individuals. Sources of protein consumption were similar between groups, except at T2 (lunch) where old individuals ingested mainly plant-based proteins compared with animal-based proteins in young and middle-aged individuals. These findings support increasing total daily protein intake on a per-meal basis in older individuals, to ensure a maximal muscle anabolic benefit is achieved.

The current RDA for dietary protein to prevent deficiencies in this macronutrient is 0.8 g·kg^−1^·day^−1^. However, despite strong evidence to suggest a benefit/need for higher protein intakes to support muscle mass and strength in old individuals (17, 18), no guidelines exist for RDA requirements in this population. Indeed, the cumulative caloric intake when consuming the RDA for the three macronutrients (protein, carbohydrates and fat) would only result in 50% of the TEI observed in the present study, with the remaining 50% of energy intake classified as *flexible* calories. Therefore, it is little surprise that the majority of young, middle-aged and old individuals in the current study achieved the protein RDA. Alternative guidelines for dietary protein intakes of 1.0-1.2 g·kg^−1^·day^−1^ have been proposed for older individuals (8). Herein, we report that 40% of young and 65% middle-aged and old individuals, respectively, did not reach a protein intake of 1.0 g·kg^−1^·day^−1^ on all 3 measurement days, clearly emphasizing the need for novel strategies to increase daily protein intake. The positive association between TEI and dietary protein intake in old individuals reported here and elsewhere (19), lends to the idea that increasing TEI would increase protein intake. Important to note here, is that we did not observe any difference in TEI between groups. Thus, whilst increasing TEI may increase dietary protein delivery for old individuals, this may come at the expense of an increase in body fat mass. Furthermore, increasing TEI is likely difficult to achieve for many old individuals, due to the well-described anorexia of aging (20). Instead, altering the composition of the *flexible* calories in favor of protein consumption might be a prosperous strategy to maintain muscle mass in old age.

The distribution pattern of daily protein intake has been proposed to distinctly stimulate MPS, with an evenly spread protein intake thought to enhance daily net postprandial muscle anabolism compared with an uneven intake pattern (21). However, others have challenged this idea (22, 23), proposing that the quantity of per-meal protein, rather than intake pattern *per se*, is key for maximizing muscle anabolism in old individuals (21, 24). The concept of an age-related impairment in the muscle anabolic response to protein provision was demonstrated by Moore et al. (10), who showed that maximal MPS stimulation occurs with the ingestion of 0.24 g·kg^−1^ in young and 0.40 g·kg^−1^ in old individuals. Comparing present study results against the threshold values for maximal MPS stimulation revealed that a majority of young individuals did not reach their threshold at T1 and T2 on all three measurement days, whilst most old individuals failed to reach their threshold at all eating occasions. Therefore, it is plausible that the dietary protein habits of our old cohort are insufficient to support skeletal muscle mass due to a failure to maximally stimulate MPS with every meal, as opposed to the uneven pattern of intake *per se*. These data are consistent with observations in British (11), Dutch (19), and US (25) cohorts of older individuals of varying health status. Taken together, these findings support calls for future studies to investigate whether increasing per-meal protein intakes in older individuals, particularly at breakfast and lunch, could maintain skeletal muscle health. Whether this is best achieved through protein supplementation, fortifying commonly consumed foods with protein/leucine, or altering meal macronutrient composition in favor of protein, remains to be elucidated.

A paucity of studies of muscle protein metabolism in middle-aged individuals, and absence of any direct comparisons of MPS against young or old individuals, make it difficult to formulate a specific MPS stimulatory threshold for this population. However, evidence shows that the postprandial MPS response to lower-dose protein ingestion does not increase above postabsorptive rates in middle-aged individuals (26, 27), similar to reported values in old individuals. Indeed, we recently reported that muscle anabolic resistance is an inevitable part of chronological aging, exacerbated by aspects of biological aging [i.e., inactivity, obesity (28)]. Thus, one might expect the MPS stimulatory threshold to fall somewhere between values for young and old individuals (0.24–0.4 g·kg^−1^) (10). Therefore, if we apply a hypothetical mid-point value of 0.32 g·kg^−1^ of dietary protein for maximal postprandial MPS stimulation in our middle-aged cohort, the proportion of individuals reaching this threshold is 5, 15, and 50% at T1, T2, and T3, respectively. However, this conjecture requires clarification through direct comparison of postprandial dose-response MPS rates between young, middle-aged and old individuals. Notwithstanding, an increase in dietary protein at breakfast and lunch in middle-aged individuals, might likely assist in the maintenance of muscle mass with advancing age.

Considering the purported threshold for maximal MPS stimulation is based on studies feeding isolated proteins, it is likely that a higher threshold for MPS saturation exists in the context of a mixed meal containing additional macronutrients. Indeed, it has been suggested that that there is no maximal anabolic response to increasing intakes of dietary protein. Specifically, increasing levels of protein intake have been shown to result in greater suppression of MPB, even when MPS is saturated (29). The suppressive effect of dietary protein on MPB is thought to be mediated by insulin secretion, with a lesser contribution of postprandial insulin directed toward MPS (30). Given that postprandial insulin-mediated regulation of muscle protein turnover may be impaired with aging (31, 32), it is possible that increasing protein consumption beyond the point of MPS saturation in older individuals might facilitate greater net muscle protein accretion through attenuating MPB. This suppressive effect of EAA on MPB is even observed beyond the suppressive effects of insulin. By further increasing intracellular EAA concentrations through protein ingestion, the additional support of MPB in regards to providing EAA precursors becomes more and more obsolete (33). However, it remains to be seen whether very high per-meal dietary protein intake (≥0.4 g·kg^−1^) leads to greater muscle mass retention in the long-term in middle-aged and old individuals.

The consumption of high-quality protein sources within the diet is essential for a robust increase in MPS (34). High-quality proteins, reflected by superior digestible indispensable amino acid scores, have a greater EAA-to-NEAA ratio, and a favorable EAA profile which closely matches the bodily needs (34). Furthermore, proteins that exhibit fast absorption and digestion kinetics, allow for a more rapid rise in circulating AAs (12). Finally, high-quality proteins have a greater protein density. Based on these characteristics, animal, rather than plant-based proteins are generally considered to be higher quality (19). In the present study, no differences were observed between groups in protein sources consumed at each meal, with the exception of lunch, where old individuals consumed mainly plant-based proteins. Lower quality proteins often exhibit an AA profile which is deficient or lacking in one or more EAA, making it crucial to combine different protein sources to provide a full complement of EAA to facilitate MPS stimulation (35). Noteworthy, is that no two plant-based proteins will be truly complementary as most plant proteins lack lysine (35). Failing to achieve a well-balanced AA profile will render the deficient protein rate-limiting in the muscle building process, as all AA are required to synthesize skeletal muscle (36). Substituting lower- for higher-quality proteins, particularly at lunch, may therefore help to support skeletal muscle maintenance in older age.

It is important to acknowledge several limitations of our study. It has been suggested that there may be potential sex-specific differences in the amount and pattern of dietary protein intake, with reports of lower protein intakes in old compared with younger men, and an opposing trend in women (37). In contrast, our data revealed no discernible difference in the amount, pattern, or source of dietary protein between men and women across age-ranges, hence why data were pooled for analysis. However, given the inherent degree of variability in all dietary protein parameters, we acknowledge that we may have been underpowered to detect significant differences between sexes. Secondly, we did not assess the physical activity status of our participants. This is important to highlight as physical activity status may be an important determinant of muscle anabolic responsiveness, particularly in older individuals (28). Specifically, physical activity/exercise acts in synergy with dietary protein ingestion to further enhance MPS (38, 39), and can therefore improve muscle anabolic responsiveness in older, and mainly frail older, individuals regularly failing to consume adequate daily protein amounts (40). Indeed, it is widely accepted that combining dietary protein strategies with regular physical activity, particularly in the form of structured resistance training, offers the most potent non-pharmacological means of maintaining or improving muscle mass, strength and function in older age (41, 42). Thirdly, our findings are only generalizable for healthy, community-dwelling older individuals, and provide little insight into the dietary protein requirements for sub-populations of older individuals with compromised muscle mass/function (for example, in frail, institutionalized, or hospitalized). Nevertheless, a recent study exploring habitual dietary protein intakes in 1051 free-living Irish individuals aged 18–90 years found comparable protein intakes and patterns to our study (43). These similarities are a likely consequence of investigating a similar study population, i.e., healthy and free-living. When comparing our results against the U.K. National Diet and Nutrition Survey (NDNS), we found that adults aged over 65 y included in the NDNS had a lower total energy intake (1,633 vs. 2,169 kcal) and absolute daily protein intake (67.4 vs. 83.4 g) (44). Whilst relative protein intakes are not presented in the NDNS, it is safe to assume that they would be lower based on the average body mass as measured by the NHS Health Survey for England 2018 (86.1 and 81.1 kg in males and 73.1 and 67.5 kg in females aged 66–74 years and over 75 years, respectively) (45). These discrepancies between the present results and those from national surveys could be due to study population differences. Indeed, studies have shown that protein intakes are similar between community-dwelling and frail older, but lower in institutionalized and hospitalized older individuals (46, 47), with a similar uneven pattern of intake in all groups. Given the rapid muscle atrophy that can occur with inactivity (47, 48), strategies to increase dietary protein delivery in institutionalized and hospitalized older individuals are of paramount importance. However, increasing dietary protein intake in hospitalized and/or malnourished older individuals is not always feasible. In this particular instance, a pattern whereby protein is pulse-fed might be preferred over an equally spread protein intake (49). By providing the majority of protein within one meal (>30 g of protein), MPS will be maximally stimulated and MPB potentially attenuated, contributing to an overall improved NPB. It is hence recommended to, first and foremost, ensure an adequate amount of protein during one meal rather than evenly spreading a suboptimal amount of protein over three main meals. Finally, it is important to acknowledge that the thresholds for maximal MPS used herein are based on a retrospective analysis and, hence, not originally intended for comparing the maximal MPS between young and old participants.

In summary, the majority of young, middle-aged and old individuals in our study met or exceeded current protein intake recommendations, even though relative and absolute protein intakes were lower in old compared with young individuals. Whilst TEI did not differ between groups, this was positively correlated with relative protein intake across age-ranges. Protein distribution throughout the day was uneven and inadequate to reach the proposed threshold for maximal MPS stimulation in old, and potentially in middle-aged individuals. Protein sources ingested at each main meal were similar across age-ranges, except for lunch where old individuals mainly consumed lower-quality plant-based proteins. Increasing protein intake, particularly at breakfast and lunch, in combination with regular physical activity/exercise in middle-aged and older individuals could potentially mitigate age-related muscle loss.

## Data Availability Statement

The datasets generated for this study are available on request to the corresponding author.

## Ethics Statement

The studies involving human participants were reviewed and approved by University of Birmingham Research Ethics Committee. The patients/participants provided their written informed consent to participate in this study.

## Author Contributions

BS, CG, and LB designed the study and wrote the manuscript together and are the guarantors of this work who will take responsibility for the integrity and accuracy of the data analysis. BS and LB carried out data collection and analysis. All authors gave their final approval of the version of the article to be published.

### Conflict of Interest

The authors declare that the research was conducted in the absence of any commercial or financial relationships that could be construed as a potential conflict of interest.
